# Measuring the psychological restorative quality of urban spaces: a vision language model-based method

**DOI:** 10.1038/s41598-026-43360-8

**Published:** 2026-04-05

**Authors:** Haoran Ma, Mei-Po Kwan

**Affiliations:** 1 Institute of Space and Earth Information Science, The Chinese University of Hong Kong, Fok Ying Tung Remote Sensing Science Building, Shatin, Hong Kong SAR China; 2 Department of Geography and Resource Management, The Chinese University of Hong Kong, Wong Foo Yuan Building, Shatin, Hong Kong SAR China

**Keywords:** Visual language model, Restorative quality, Urban spaces, Street view images, Attention Restoration Theory, Environmental social sciences, Psychology, Psychology

## Abstract

Well-designed urban environments crucially mitigate stress and enhance mental well-being through their restorative qualities. However, subjective surveys lack spatial scalability, while objective machine learning often fails to capture the complexity of human perceptual experiences. To address this gap, this study proposes a hybrid framework based on Vision Language Models (VLMs) and human experiences to assess the restorative quality of urban spaces. Using Shenzhen, China as a case study, we gathered subjective and objective knowledge from PRS-11 surveys and ChatGPT-4 descriptions of 566 street view images, incorporating this into VLM prompts via the Contrastive Language-Image Pretraining (CLIP) model. Through prompt engineering, the VLM evaluated 2,224 additional images, with semantic networks analyzing the decision-making process. Results demonstrate that: (1) our method significantly outperformed Random Forest, with an R² increase of 0.535 attributed to prior knowledge fusion; (2) restorative quality exhibits spatial heterogeneity, clustering in developed districts near park and coastal zones; and (3) semantic network analysis further revealed the decision rationales of VLMs across different restorative dimensions, providing design guidelines for low restorative quality spaces. This research offers a novel methodology for assessing restorative quality of urban spaces, providing practical tools for sustainable development and human mental well-being.

**Supplementary Information:**

The online version contains supplementary material available at 10.1038/s41598-026-43360-8.

## Introduction

### The role of urban space in residents’ mental health

Urban residents worldwide currently face multiple pressures, including work stress, living costs, and social competition, leading to increasingly severe mental health issues^1–3^. Residents in Chinese first-tier cities are no exception. Numerous surveys have revealed alarming rates of mental health problems among this group^4–6^. Moderate to severe mental issues are widespread and have become critical factors affecting career development^7^ and even safety^8^. The Report on the Mental Health Development of Chinese Citizens (2023–2024) identifies high housing prices, work pressure, and future uncertainty as primary stressors^1^. It also highlights significant group differences, such as a higher risk among residents in first-tier cities. Notably, over 70% of young urban residents show significantly elevated anxiety risk. Effective intervention pathways are urgently needed to address these issues.

As the core setting for residents’ daily activities, the urban environment is a significant factor influencing mental health^9^. Well-designed urban spaces can effectively promote residents’ mental health and well-being by providing restorative experiences^10–12^. Multiple studies have confirmed that factors such as urban green space ratio^13^, vegetation richness^14^, green space visibility^15^, spatial diversity^16^, canopy coverage^17^, and biodiversity^18^ are significantly associated with urban residents’ mental health. Conversely, urban environments lacking natural elements can increase resident stress levels, reduce opportunities for mental restoration, and potentially lead to mental health issues like depression and anxiety^19,20^. These findings collectively underscore the core value of urban spaces as a preventive intervention and developmental platform for promoting residents’ mental health.

### Restorative effect of urban spaces and assessment challenges

The positive benefits of urban spaces for mental health are primarily achieved through their restorative properties^21,22^. The Stress Reduction Theory (SRT) and Attention Restoration Theory (ART) from environmental psychology provide the theoretical foundation for this. SRT posits that humans possess an innate, genetically-based need for natural environments; this instinct enables them to remain calm and effectively reduce physiological and psychological stress when exposed to nature^23–25^. ART suggests that prolonged states of intense “directed attention” in daily life lead to mental fatigue, while natural environments offer attractions for “involuntary attention,” allowing the brain to rest and recover^26^. ART emphasizes four core elements of restorative environments^27^: “being away,” “fascination,” “extent,” and “compatibility.” For instance, fascination refers to an environment effortlessly capturing our attention, allowing the brain to rest and thereby restoring directed attention^28,29^. However, accurately and objectively assessing the restorative quality of urban spaces on a large scale presents significant challenges.

Traditional methods for assessing the quality of an environment for psychological restoration primarily rely on subjective reports, such as questionnaires^30,31^ and interviews^32,33^. The commonly used scale includes the Perceived Restorativeness Scale (PRS), which has 26 items^34^, and a simplified version, the PRS-11, which has 11 questions^35^. However, these subjective assessment methods suffer from inherent limitations. Questionnaire results, for example, can only roughly describe one aspect of psychological state rather than the whole picture^36^. Subjectivity is unavoidable, potentially leading to missing information and questions about data reliability^37,38^. Furthermore, these methods often involve small sample sizes, high costs, poor spatiotemporal scalability, and struggle to capture nuanced differences in spatial restorative quality across an entire urban spaces^39–41^. Fortunately, the emergence of street view images (SVIs) combined with computer vision techniques offers new opportunities for large-scale assessment of spatial restoration quality^42–44^. Research utilizing methods like semantic segmentation extracts environmental features (e.g., vegetation proportion) from SVIs and employs machine learning to assess their psychological impacts^45^. Recently, some studies based on street view data have begun exploring the influence of different visual-level environmental features^10^, perceived distance^46^ and spatial structures^16^ on psychological restoration.

Despite significant progress, street view image-based assessment methods still exhibit notable limitations. The most prominent issue is that machine learning methods often operate as “black boxes"^47^. While capable of predicting urban space restoration quality and revealing the influence of environmental elements on predictions, these machine-centric approaches neglect the role of human perception in the decision-making process. Specifically, restoration theory posits that mental restoration is a dynamic psychological process of perception-stimulus-restoration^48^. Individuals perceive the urban space instantaneously through senses like vision, forming an initial place schema. Then, specific elements within the space (e.g., green) act as stimuli, triggering emotional appraisal and cognitive processing by the individual, subsequently activating “restorative perception”–the individual’s subjective judgment of whether the space can alleviate mental fatigue and restore attention. Finally, restorative perception modulates subsequent behavioral tendencies^49,50^. However, most machine learning methods struggle to fully capture this dynamic subjective experience, as they typically map street view pixels directly to restoration quality levels without explicitly modeling the underlying perceptual and cognitive stages.

### Opportunities and disadvantages presented by visual language models

The potential of Large Language Models (LLMs) to simulate human behavior and substitute human participants in experiments represents a new perspective for overcoming the limitations of existing assessment methods^51–53^. For instance, Stanford’s Generative Agents project successfully created a virtual society, populated by 25 AI agents powered by LLMs. Through three core mechanisms–memory, reflection, and planning–these agents spontaneously conduct daily life, build relationships, and simulate complex group behaviors and information dissemination^54^. Visual Language Models (VLMs), as a crucial branch of LLMs, present a core opportunity through their powerful multimodal fusion capability^55,56^. Previous research has preliminarily explored using VLMs to assess environmental preferences, urban walkability, and automated environmental audits. In studies on environmental preference, Tung et al.^57^ found that GPT-4 and LLaVA showed high agreement with human judgments on complexity, coherence, mystery, and overall preference, though agreement on legibility was inconsistent. In urban walkability assessments, scores aligned with human ratings in 80%–90% of cases across four out of five trials. ChatGPT captured not only typical street view elements like greenery, sidewalks, and roads but also finer details like bollards and handrails, while holistically assessing walkability by considering contextual relationships between objects in the image^58^. These studies confirm the validity of using VLMs for assessing meso-scale and micro-scale environmental features and the feasibility of simulating human environmental perception^59^.

Despite these encouraging advances, current VLMs still exhibit significant limitations. The primary challenge lies in the models’ inherent reliance on statistical patterns within training data, making it difficult to fully capture the subjectivity, contextual dependencies, and value judgments inherent in human experience. Furthermore, VLMs demonstrate inadequacies in processing subtle variations in urban environmental elements, complex spatial relationships, and the dynamic interactions between physical features and human perception^60^. These limitations underscore the importance of integrating human survey data as prior knowledge into model assessments, particularly for highly personalized and culturally sensitive evaluation tasks such as environmental preferences. Initial attempts to address these limitations have shown promise, with researchers incorporating survey data as anchor points into VLMs’ decision processes^61^. These integrations have demonstrated significant improvements in model prediction accuracy and consistency with human assessments. Therefore, a hybrid framework incorporating human experiential knowledge into VLMs could potentially enhance the accuracy of restorative perception simulation in this study.

Based on the aforementioned background, theoretical foundations, limitations of existing methods, and the new opportunities presented by LLMs, this study aims to explore the potential of VLMs in assessing the psychological restoration quality of urban spaces. Specifically, this research addresses the following questions: (1) Can VLMs serve as residents in assessing the restorative quality of urban spaces? (2) What are the spatial variations in restorative quality? (3) Are the VLM predictions consistent with previous research findings? (4) How do VLMs simulate human perception-stimulus-restoration process?

## Method and data

**Fig. 1:**
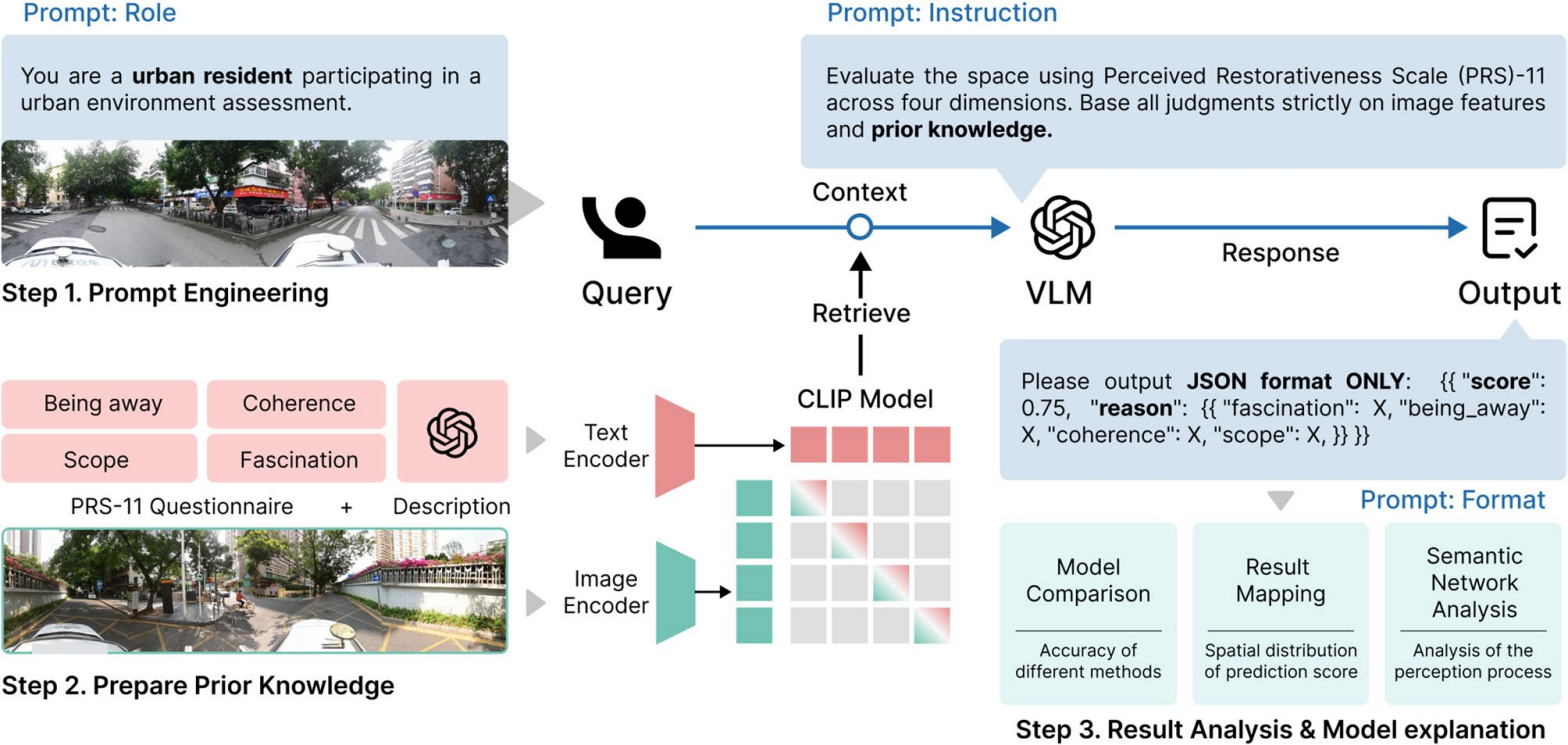
Research framework.

This study proposes a VLM-based hybrid framework for assessing the restorative quality of urban spaces (Fig. 1). Our framework comprises three key steps: prompt engineering, prior knowledge preparation, and result analysis. Specifically, during the prompt engineering phase (Step 1), a consistent and targeted evaluator perspective is ensured by assigning the role of a resident participating in urban spaces restorative quality assessment. In the prior knowledge preparation phase (Step 2), we embed textual descriptions and environmental images multimodally. The textual content incorporates the subjective scores from the PRS-11 survey, combined with the objective scene descriptions inferred by VLMs. These are then retrieved as an evaluation standard in the prompt engineering process (Step 1). Finally, results including an overall rating and specific rationales for each dimension are obtained for further analysis and model interpretation (Step 3).

### Research areas and data

**Fig. 2:**
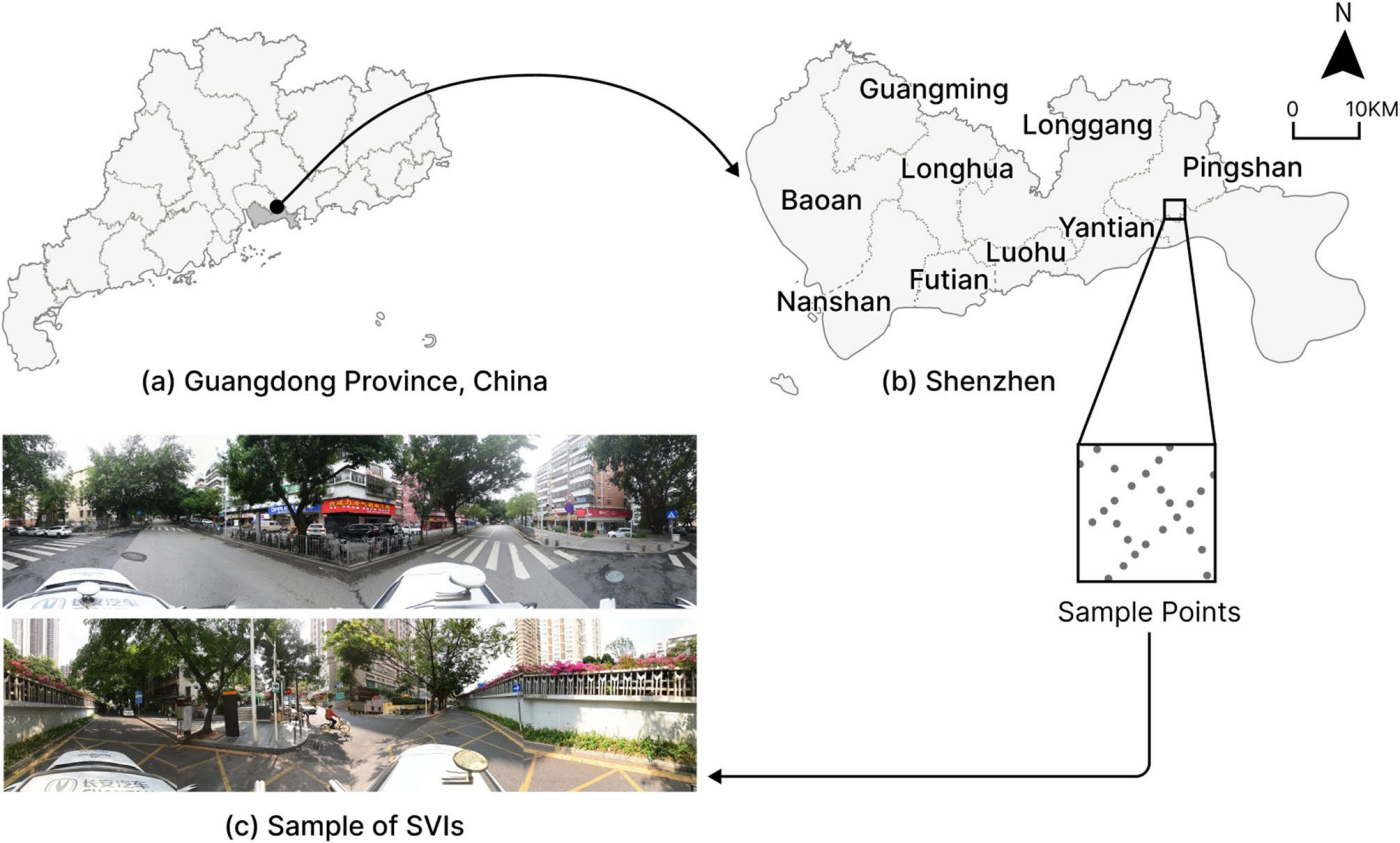
Prior knowledge preparation based on the CLIP model. Map generated using ArcGIS Pro 3.5.0 (Esri Inc., https://www.esri.com*).*

Our study focuses on Shenzhen City, Guangdong Province, China (Fig. 2). As a high-density metropolis with a population exceeding 17 million, Shenzhen faces growing mental health challenges associated with rapid urbanization and intense work-life pressures^4,62^. In recent years, the government has strengthened public mental health services, establishing community counseling centers and offering resources addressing stress, adaptation, and emotional management. However, stigma associated with seeking psychological counseling compromises the effectiveness of these mental health initiatives. Consequently, a current priority is to leverage urban planning and spatial design to promote resident’s mental well-being.

We collected street view images in June 2025 using the Baidu Map API v2 service (https://lbsyun.baidu.com/faq/api?title=viewstatic). Baidu Maps provides extensive streetview coverage of Chinese cities and is widely used in previous studies^63^. These SVIs were used for the assessment of urban spaces’ restorative quality. We first generated sampling points for street views at 200-meter intervals along the OpenStreetMap (OSM) (https://www.openstreetmap.org/) road network across Shenzhen, yielding 2932 panoramic SVIs. All images were captured during the summer season (June to September). This period was selected for data collection as the climatic conditions and seasonal foliage during summer are representative of the city’s most visually distinct phase, which is critical for assessing the restorative potential of urban spaces based on visual cues^4,64^. Subsequently, images exhibiting blurring, missing data, or color distortion were filtered out, resulting in a final dataset of 2780 panoramic SVIs. The distribution of our sample points across the entire urban spaces ensured the minimization of potential spatial sampling bias in the results.

### Prompt engineering and model selection

To accurately assess the restorative quality of urban spaces, we used prompt engineering to guide VLMs as a resident and score SVIs. Prompt engineering, also known as contextual prompting, represents an advanced approach that directs VLMs to desired outcomes through carefully designed input instructions without modifying the underlying model architecture^65^. This method offers flexibility in adapting to different participant characteristics and research scenarios compared to model fine-tuning (e.g., LoRA)^66^. An effective prompt structure typically includes four key elements: role definition, contextual background, task instructions, and output standardization. This framework ensures the consistency and relevance of model responses while maintaining computational efficiency.

For role definition, we established the model’s perspective as a resident participating in urban environment assessment (*prompt: "You are a resident participating in an urban environment assessment."*). This role assignment aimed to ensure appropriate evaluation context and perspective alignment. Then, the contextual background introduced the PRS-11 as the primary assessment tool, providing the necessary theoretical framework for the evaluation task. This scale item effectively captures the substantive effects of environmental stimuli in a semantic way^35^. For PRS-11 content, please refer to Supplementary Table S1. Task instructions were explicitly structured to guide the model through a systematic analysis process, requiring evaluation across the four key restorativeness dimensions. To ensure data consistency and facilitate subsequent analysis, we standardized the output in JSON format, incorporating both average dimensional scores and qualitative reasoning. The specific prompt can be found in Supplementary Table S2.

In addition, we referred to the LLM leaderboard (https://llm-stats.com/) and selected six VLMs as baseline models, including four closed-source VLMs (i.e., Grok-4, Claude-3.7-Sonnet, Gemini-2.5-Flash, and ChatGPT-4o) and two open-source VLMs (i.e., Qwen2.5-VL-72B, Llama-4-Maverick). These models demonstrate strong performance in image question answering and scene understanding tasks^67^. The closed-source VLMs were accessed through their respective commercial APIs, while the open-source VLMs were deployed locally using the Ollama platform. The hardware used was an NVIDIA-4090 GPU with 48GB of RAM. To ensure consistent model outputs, we standardized two key parameters: temperature (controlling output diversity, with higher values increasing randomness) and maximum tokens (limiting output length). All models were configured with max_tokens = 1000 to ensure complete outputs and temperature = 0.7 to balance human-likeness and diversity in urban spaces’ restoration quality evaluation.

### Prior knowledge preparation and utilization

The incorporation of human experiences may enhance model accuracy. To validate our hypothesis, we selected 556 SVIs (i.e., 20% of total) as our dataset using a grid-based method, with 80% allocated for training and the remaining 20% for testing through random sampling. For details about the sample selection process, please refer to Supplementary Fig. S1. We divided the preparation and utilization of prior knowledge into two phases: (1) acquisition of textual content; (2) multimodal embedding and retrieval.

**Fig. 3:**
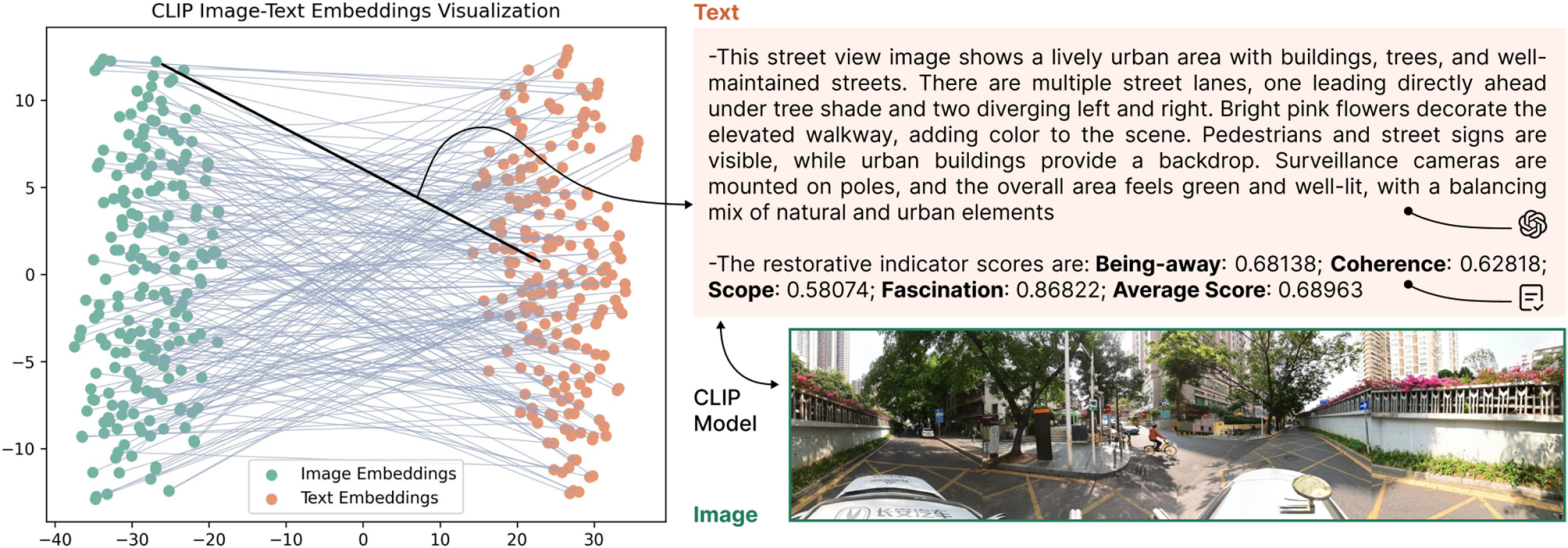
Prior knowledge preparation based on the CLIP model.

**Acquisition of textual content.** We obtained text content through a combination of subjective and objective approaches (Fig. 3). Specifically, we first obtained restorative quality evaluation results for each image based on the PRS-11 questionnaire (5-point Likert scale). We designed an assessment platform that required participants to evaluate each image pair within a 40-second time limit. Previous research has indicated that 40 s minimizes potential bias caused by participant perceptual fatigue^16^. During a one-week online survey, we collected evaluations from 122 residents aged 19–43 years (mean age 25.2 ± 5.1 years; the females averaged 24.7 ± 4.0 years and the males averaged 25.9 ± 6.0 years). The age range for participants in this study was set at 18–45 years, as this demographic constitutes the primary urban population and represents the main users of urban spaces. Each image was evaluated at least 20 times. To be consistent with the VLMs evaluation results, we normalized the scores from 0 to 1 for each dimension to facilitate subsequent analysis. Finally, we calculated the average scores across four dimensions to represent the mean restorative quality of 556 SVIs (Table 1). The ethical aspects of this experiment were reviewed and approved by the Institutional Review Board of the authors’ university.

**Table 1:** Statistical summary of restorative assessment indicators.

Dimension	Count	Mean	Std.	95% CI
Fascination	556	0.445	0.211	(0.428, 0.463)
Being Away	556	0.393	0.231	(0.373, 0.412)
Coherence	556	0.461	0.214	(0.443, 0.479)
Scope	556	0.455	0.207	(0.438, 0.472)
Average	556	0.439	0.127	(0.428, 0.449)

Subsequently, we utilized VLMs to obtain objective descriptions of 556 SVIs. Previous research has shown that ChatGPT-4 excels at capturing comprehensive meso-scale features and subtle micro-scale features. Compared to traditional computer vision, it can understand fine-grained spatial design details and contextual relationships between objects in images^68^. Therefore, we employed ChatGPT-4 as the baseline model to obtain objective descriptions of urban spaces (*prompt: "Please describe this street view image."*). We constrained the output to 150 words (approximately 200 tokens), a length determined through multiple rounds of ablation that preserves the scene’s key information such as object elements, spatial structure, and color relationships without redundancy. Finally, we combined subjective and objective content as text input for multimodal embedding (Fig. 3).

**Multimodal embedding and retrieval.** After obtaining the content of the objective environment and subjective perception, it is crucial to integrate them into the decision-making process of VLMs. In this paper, we embed prior knowledge into a shared vector space and train the model by maximizing the similarity between matched pairs. By retrieving extreme values, we can effectively match the SVI with its similar objects. This approach not only circumvents the massive data requirement of training a bespoke model from scratch but also enhances the accuracy of restorative-score estimation by leveraging a pre-constructed, sophisticated knowledge base.

To obtain high-quality multimodal feature representations, we employed the Contrastive Language-Image Pretraining (CLIP) model for joint image and text encoding. The CLIP model, pre-trained on large-scale image-text pairs, effectively understands semantic associations between image content and text descriptions^69^. In this study, we used the pre-trained CLIP-ViT-B-32 as our feature extractor, which offers good generalization ability and computational efficiency. Specifically, for each SVI, we first obtained 512-dimensional image features ${I}_{emb}$ and 512-dimensional text features ${T}_{emb}$ through the visual and text encoder, respectively. The visual encoder adopts a Vision Transformer (ViT) architecture comprising 12 transformer encoding layers^70^. Images are first divided into 32 × 32 patches, and position information is added. After linear projection, the patches are input into the transformer, where the self-attention mechanism determines the importance of each patch, according to:

1 $$Attention\left(Q,K,V\right)=softmax\left(\frac{{QK}^{T}}{\sqrt{{d}_{K}}}\right)V$$

where $Q$, $K$, and $V$ represent Query, Key and Value matrices respectively, ${d}_{K}$ is the dimension of the key vectors. Additionally, the text encoder employs a transformer architecture with 12 encoding layers to process tokenized text sequences. Both encoders are trained jointly, optimizing cross-modal feature space through contrastive learning.

In addition, we performed cross-modal retrieval through cosine similarity calculations. We applied $L2$ normalization to feature vectors and used matrix multiplication for batch similarity calculations, returning the top-$k$ results with the highest similarity, calculated as:

2 $$similarity\left( {I_{{emb}} ,{\mathrm{~}}T_{{emb}} } \right) = \frac{{I_{{emb}} \cdot{\mathrm{~}}T_{{emb}} }}{{\left| {\left| {I_{{emb}} } \right|} \right|\cdot{\mathrm{~}}\left| {\left| {T_{{emb}} } \right|} \right|}}$$

where ${I}_{emb}$ represents the query image feature vector and ${T}_{emb}$ represents the candidate text feature vector. By calculating the similarities between the query image and all candidate texts, we can retrieve text descriptions semantically closest to the query image. In the specific experiments, we retrieve the top-$K$ pieces of prior knowledge based on similarity. When $K=2$, it means that two similar results, each comprising a subjective rating and its corresponding objective description, are incorporated into the decision-making process of the VLMs. Each piece of prior knowledge contains approximately 160 words, which corresponds to about 230 tokens. We visualized the spatial positions and matching relationships of image-text pairs using principal components analysis (PCA) dimensionality reduction (Fig. 3). These results serve as prior knowledge input to VLMs through prompt engineering, providing a basis standard for accurate evaluation of urban spaces’ restorative quality.

### Results evaluation and model explanation

To comprehensively evaluate model performance and gain an in-depth understanding of the prediction mechanisms, this study employs a combined quantitative and qualitative analytical approach. For quantitative assessment, we adopted Mean Squared Error (MSE), Root Mean Squared Error (RMSE), and R² as the primary evaluation metrics^71^. MSE and RMSE are defined respectively as:

3 $$\begin{array}{c}\mathrm{M}\mathrm{S}\mathrm{E}=\frac{1}{n}{\sum}_{i=1}^{n}{\left({y}_{i}-\widehat{{y}_{i}}\right)}^{2}\end{array}$$

4 $$\begin{array}{c}\mathrm{R}\mathrm{M}\mathrm{S}\mathrm{E}=\sqrt{\frac{1}{n}{\sum}_{i=1}^{n}{\left({y}_{i}-\widehat{{y}_{i}}\right)}^{2}}\end{array}$$

where ${y}_{i}$ represents the true value of the $i$th sample, $\widehat{{y}_{i}}$ represents the corresponding predicted value, and $n$ is the total number of samples. MSE reflects the average squared deviation between predicted and true values. Smaller values of MSE and RMSE indicate better predictive performance. The R² value ranges from [0, 1], with larger values indicating a better model fit.

In addition, we also compared the performance of traditional machine learning-based methods in this study. Specifically, we first obtained visual features at different levels, including pixel-level, perceptual-level, and semantic-level. These features have been well-validated in previous studies for their impact on spatial restorative quality^10^. After further removing missing values and elements that did not conform to the urban context, we finally retained 24 variables. The variable statistics are detailed in Supplementary Table S3. We then trained five machine learning models, including Random Forest (RF), Decision Tree (DT), Gradient Boosted Decision Trees (GBDT), K-Nearest Neighbors (KNN), and Linear Regression (LR)^71^, with 80% of the data used as the training set and the remaining 20% as the test set. To minimize result bias, we ensured that the parameters of each model were consistent.

In terms of qualitative analysis, we used semantic network analysis to interpret the decision-making process of the model. By constructing a semantic association network, we can reveal the intrinsic connections between different environmental elements and their impact mechanisms on restorative quality^72^. Specifically, we first extracted structured triplets from the evaluation texts generated by VLMs using ChatGPT-4, which has strong text understanding capabilities^73^. The construction follows the cognitive framework of perception-stimulus-restoration^48^. The detailed prompt for extracting triplets can be found in our code (https://github.com/MMHHRR/VLM_restorative-quality). We considered the spatial elements (e.g., buildings, trees) as the perception sources that shape environmental experiences, psychological perceptions (e.g., a sense of calm) as the stimuli that trigger responses, and behavioral reactions (e.g., escape) as the restoration outcomes of this process.

We constructed independent semantic networks based on the four dimensions of restorativeness, with each dimension’s network containing three types of connections: positive (promoting restorative quality), negative (inhibiting restorative quality), and neutral connections (no clear tendency). Second, to ensure the reliability of the triplets extracted by ChatGPT-4, we conducted rigorous, independent validation and contextual evaluation of the extraction results manually. Finally, we analyzed four core indicators of the semantic network, including the number of nodes and edges, average degree, and the proportion of positive, negative, and neutral connections^74^. The average degree is calculated as:

5 $$\stackrel{-}{k}=\frac{2L}{N}$$

where $\stackrel{-}{k}$ is the average degree, $L$ is the total number of edges in the network, and $N$ is the total number of nodes. Additionally, by identifying the top 20 nodes with the highest degree centrality in each dimension, we explored the key mechanisms for spatial restorative quality formation. The degree centrality of a node is calculated as:

6 $${C}_{D\left({v}_{i}\right)}=\frac{d\left({v}_{i}\right)}{N-1}$$

where ${C}_{D\left({v}_{i}\right)}$ is the degree centrality of node ${v}_{i}$, $d\left({v}_{i}\right)$ is the number of edges directly connected to node ${v}_{i}$, and $N$ is the total number of nodes in the network. This semantic network-based interpretation method not only verifies existing theoretical assumptions but also helps to discover new knowledge patterns, providing empirical evidence for urban space design.

## Results

### Comparison of model performance and methods

We conducted a comparative analysis of the performance between traditional and VLM-based methods in assessing the restorative quality of urban spaces (Table 2). The results show that VLM-based methods significantly outperformed the traditional method. Within our framework, Claude-3.7-Sonnet exhibited the best performance among the VLMs (MSE = 0.003, RMSE = 0.061, and R² = 0.760). Also, our approach outperformed the best-performing traditional method (RF) with a 0.535 performance enhancement in R². Followed by Grok-4, Gemini-2.5-Flash and ChatGPT-4o, which also demonstrated better performance, with R² of 0.679, 0.627, and 0.560, respectively. Among the traditional methods, RF performed best, but with a significantly lower R² of 0.275 compared to VLM-based methods. The performance of other ML methods, such as DT, GBDT, KNN and LR, was relatively weak, with an R² value below 0.230. It is noteworthy that the open-source VLMs (e.g., Qwen2.5-VL-72B and Llama-4-Maverick), while underperforming commercial models, still surpassed traditional methods. These results highlight the advantages of VLM-based methods for assessing the restorative quality of urban spaces.

**Table 2:** Model performance.

Method	Model	MSE↓	RMSE↓	*R*^2^↑
ML	RF	0.112	0.334	0.225
	DT	0.124	0.352	0.033
	GBDT	0.112	0.335	0.211
	KNN	0.121	0.348	0.083
	LR	0.114	0.337	0.193
VLM (Our)	Grok-4	0.005	0.071	0.679
	Claude-3.7-Sonnet	0.003	0.061	0.760
	Gemini-2.5-Flash	0.006	0.076	0.627
	ChatGPT-4o	0.006	0.083	0.560
	Qwen2.5-VL-72B*	0.010	0.101	0.342
	Llama-4-Maverick*	0.011	0.105	0.291

* represents open-source models and the underline indicates the optimal model.

**Fig. 4:**
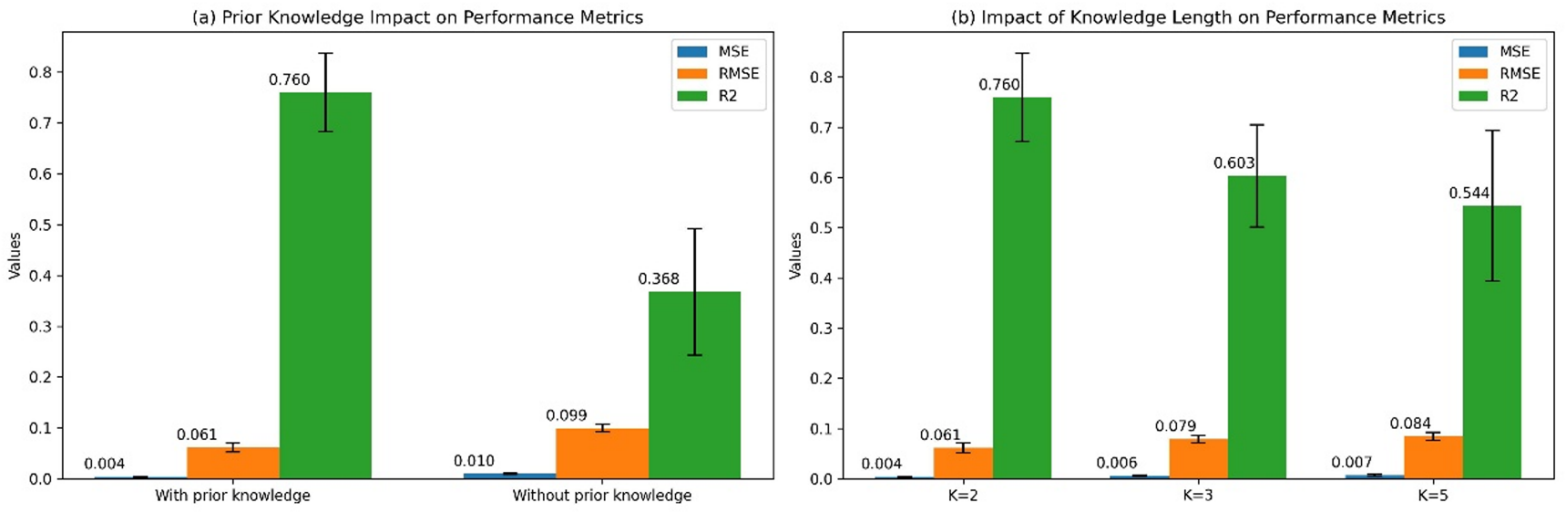
Ablation experiments based on different prior knowledge strategies and their length. (**a**) Impact of prior knowledge on model performance. (**b**) Impact of the length of prior knowledge on model performance.

To identify the factors influencing model performance, we conducted two ablation studies based on Claude-3.7-Sonnet model, focusing on the effect of prior knowledge and its length on performance (Fig. 4). Specifically, the introduction of prior knowledge significantly enhanced model performance by 0.392 in R² (Fig. 4a). We found that the model achieved optimal performance when prior knowledge was used (MSE = 0.004, RMSE = 0.061, R² = 0.760). Conversely, performance deteriorated significantly without prior knowledge (MSE = 0.010, RMSE = 0.099, R² = 0.368), which confirms the crucial role of prior knowledge in improving performance. Additionally, varying the length parameter $K$ of the prior knowledge revealed that concise, refined prior knowledge enables the model to accurately assess the psychological restorative quality of urban spaces (Fig. 4b). Specifically, the model performed best at $K=2$ (the shortest prior knowledge length), with an R² of 0.760. As the length of the prior knowledge increased, model performance showed a declining trend, with the R² decreasing to 0.603 at $K=3$ and further dropping to 0.544 at $K=5$. The MSE and RMSE metrics exhibited similar trends, gradually worsening with increasing $K$ values. This suggests that excessively long prior knowledge may introduce noise, thereby impairing the judgment capabilities of the model.

### Mapping of the restorative quality of urban spaces

**Fig. 5:**
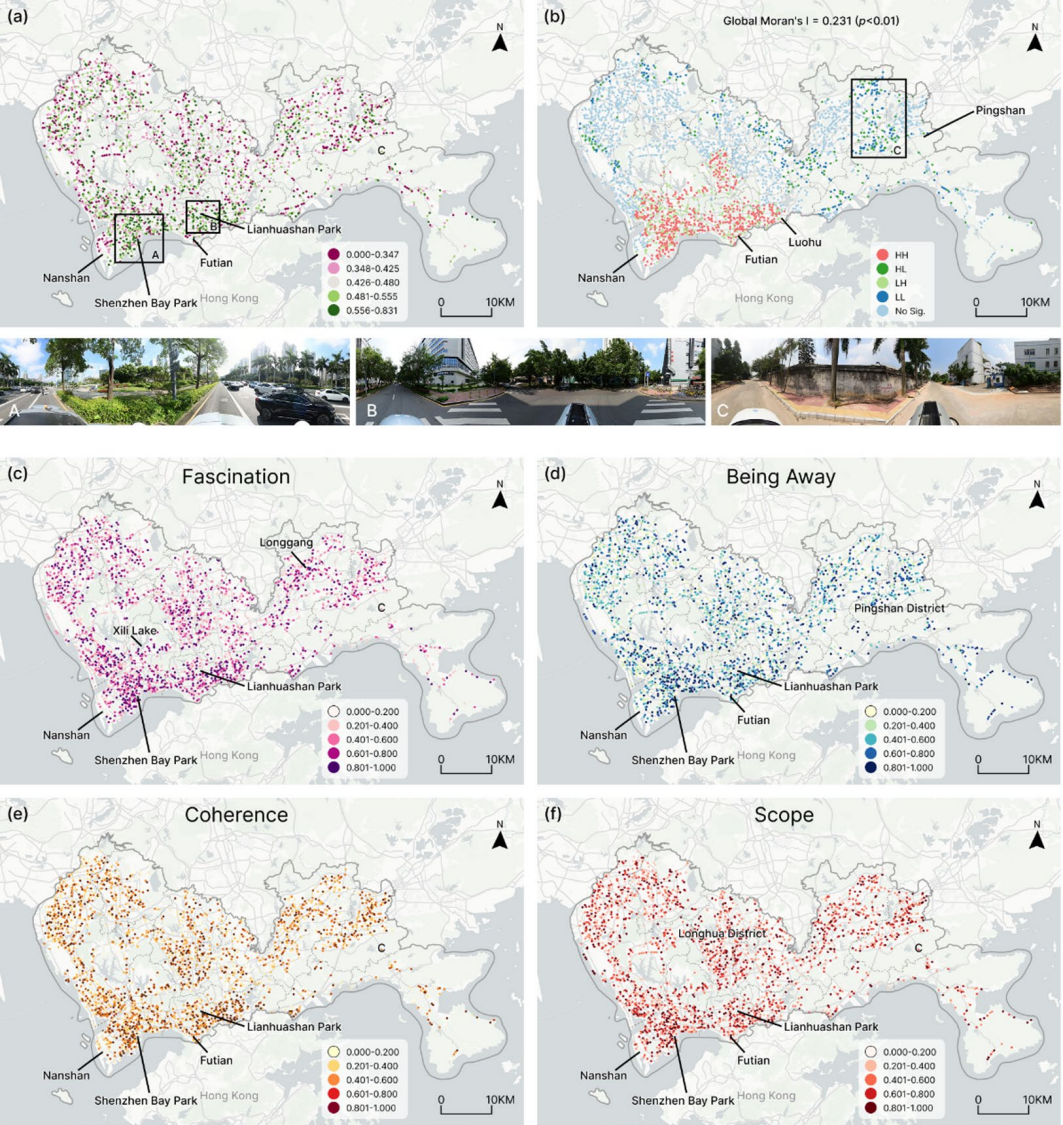
Prediction results mapping and analysis. (**a**) Mapping of prediction results. (**b**) Spatial pattern analysis using local Moran’s I. (**c**-**f**) Four dimensions of restorative quality. Map generated using ArcGIS Pro 3.5.0 (Esri Inc., https://www.esri.com*).*

**Fig. 6:**
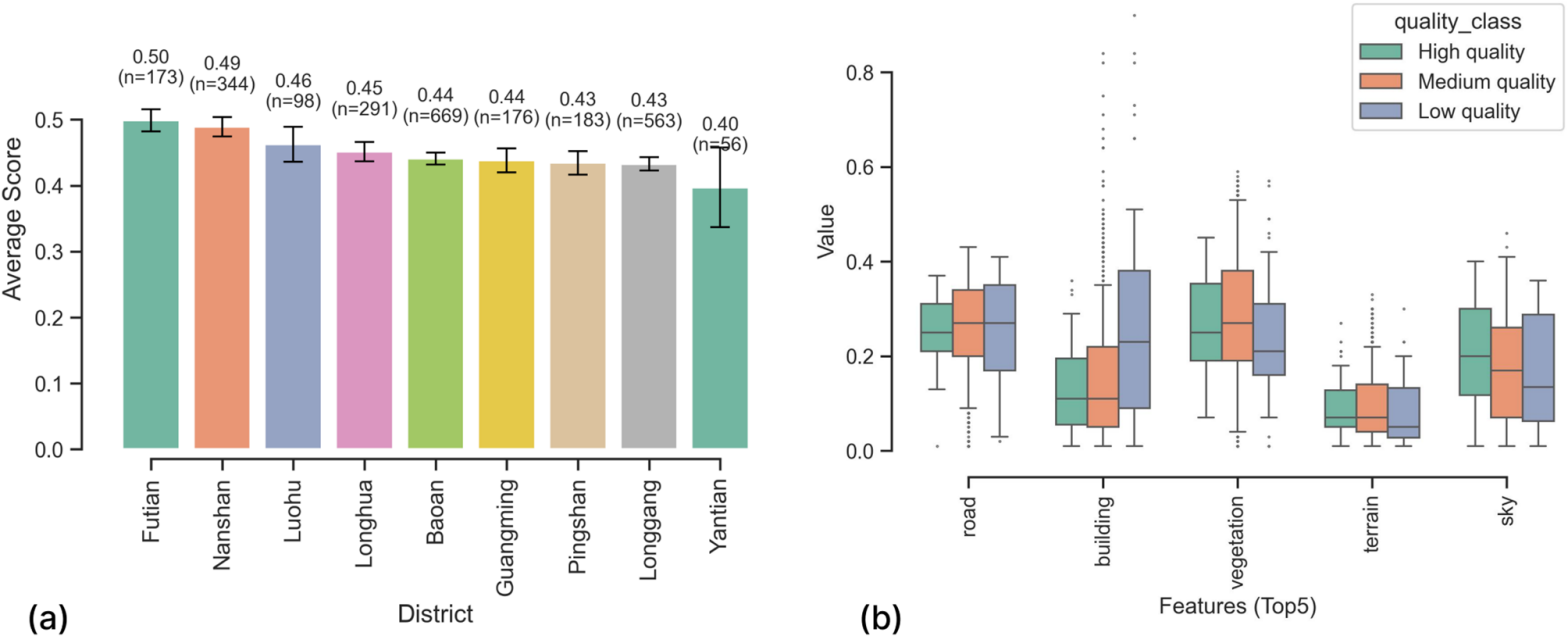
Distribution of average restorative scores by district (a) and visual difference between different levels of restorative quality (b).

Based on the Claude-3.7-Sonnet model, we predicted the restorative quality of the remaining 2,224 urban spaces. As shown in Fig. 5a, spaces with high restorative quality were primarily concentrated in districts such as Nanshan, Futian, and Luohu. Among these, Futian District exhibited the highest overall restorative quality score of 0.50, followed by Nanshan District with a score of 0.49 (Fig. 6a). The superior restorative quality in these two districts may be attributed to the local government’s emphasis on urban greening and the development of public spaces. Notable examples include Lianhuashan Park in Futian District and Shenzhen Bay Park in Nanshan District, which not only have high green coverage rates but also prioritize user experience in their spatial design, providing pleasant landscape views and comfortable recreational environments (Fig. 5c). Conversely, in newly developed areas such as Pingshan District, the expansion of industrial zones and infrastructure construction has consumed substantial land resources, leading to a reduction in original natural vegetation coverage. In such a development context, the Fascination and Being Away dimensions are lower than those in other areas (Fig. 5c–d). Furthermore, Fig. 5b presents the results of spatial autocorrelation analysis (global Moran’s I = 0.231, *p* < 0.01), indicating significant spatial clustering characteristics of restorative quality. This spatial distribution pattern suggests that areas with specific functional attributes and environmental elements are more conducive to creating psychologically restorative environments.

Additionally, to further understand the differences in urban spaces influencing varying restorative quality levels, we categorized them into three classes (High, Medium, and Low restoration quality) based on percentiles and compared the differences in visual proportions of five key spatial elements (Fig. 6b). These features are obtained through semantic segmentation based on the MaskFormer model. This model can segment 150 object categories and has been widely used in urban science research due to its superior performance compared to other models^75^. Results indicated that artificial elements (e.g., roads, buildings) had the highest visual proportion in low-quality restorative spaces (bottom 25%). In contrast, natural elements (such as vegetation, terrain, and sky) showed the highest visual proportions in high restorative quality spaces (top 25%). These differences align with previous research findings, suggesting that urban spaces with prominent natural elements offer greater psychological restoration benefits. This also validates the reliability of environmental features identified by VLMs during the assessment process.

**Fig. 7:**
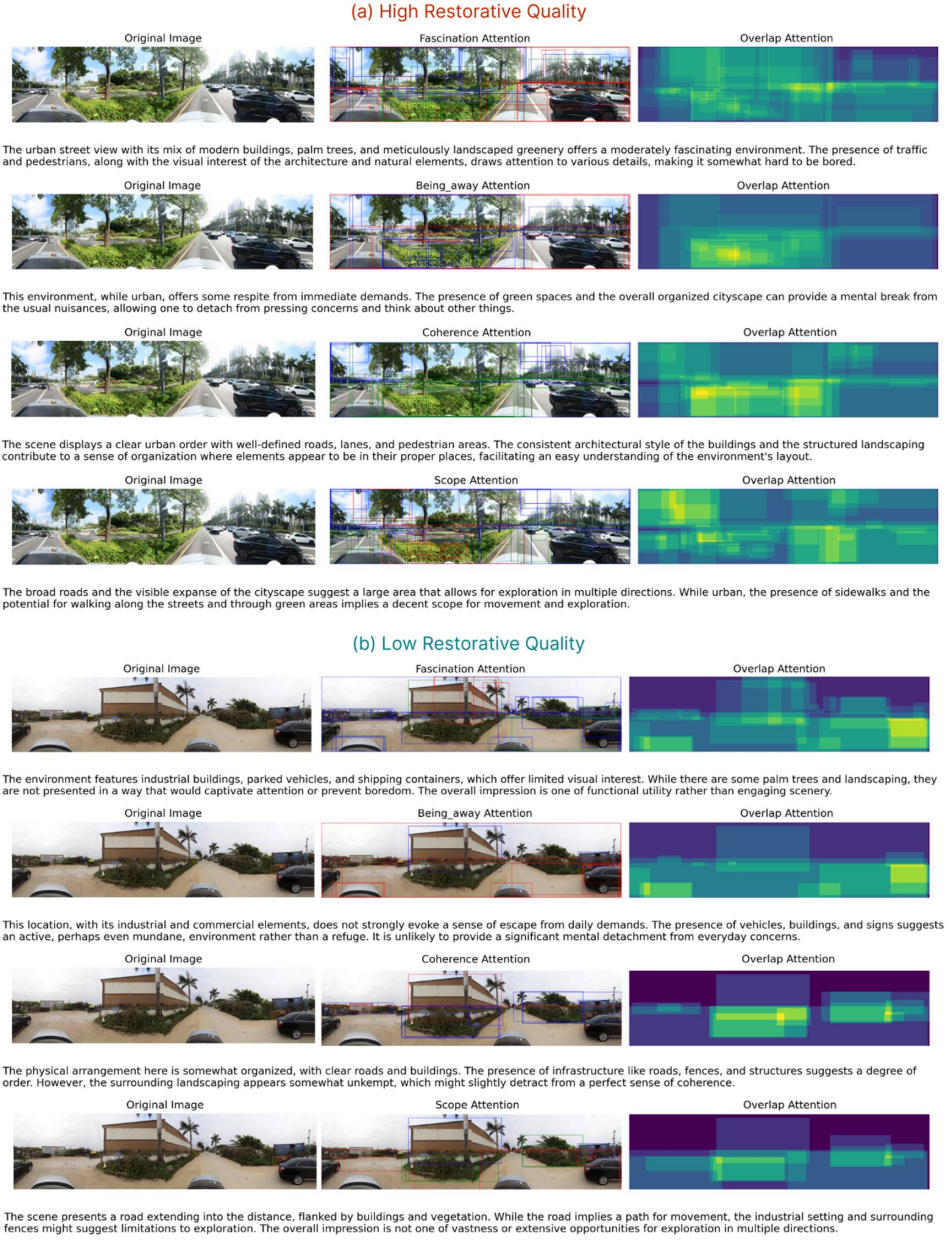
Sematic attention of each dimension. (**a**) Pseudo-CAM of a high restorative quality space. (**b**) Pseudo-CAM of a low restorative quality space.

We utilized the explanatory text generated by the VLMs and employed the CLIP model to annotate corresponding elements within the images. We then plotted the bounding boxes of these identified elements and visualized their overlapping frequency as pseudo-class activation maps (CAMs), as shown in Fig. 7. For instance, within high restorative quality spaces for the Coherence dimension, the VLMs predominantly focused on the organization of roads and the orderly arrangement of street trees on both sides. This finding further elucidates the specific objects that VLMs prioritize during their decision-making process across different dimensions, thereby enhancing the credibility and interpretability of the results.

### Semantic network analysis of VLM-based restorative assessment

To further understand the VLM-generated assessment text, we modeled and analyzed it using semantic networks. Network analysis (Table 3) revealed distinct characteristics across the four dimensions (Fascination, Being Away, Coherence, Scope). The Scope dimension exhibited the highest average degree (6.517), indicating dense connections among nodes related to spatial extent and navigability. The Being Away dimension showed the highest number of nodes (1,445) but a relatively low average degree (5.977), suggesting that feelings of escape may arise from diverse, and relatively independent environmental factors. Notably, the Coherence dimension had the highest proportion of positive restorative connections (60.0%), significantly exceeding the other dimensions, potentially reflecting a high level of overall urban spatial coherence.

**Table 3:** Semantic network analysis of four restorative dimensions.

Dimension	Nodes	Edges	Degree (Avg.)	Positive (%)	Negative (%)	Neutral (%)
Fascination	1481	4679	6.318	38.0	47.1	15.0
Being away	1557	4777	6.136	19.4	71.3	9.3
Coherence	1445	4319	5.977	60.0	34.1	6.0
Scope	1029	3353	6.517	49.0	40.0	11.0

A positive edge is one that contributes to high restorative quality.

**Fig. 8:**
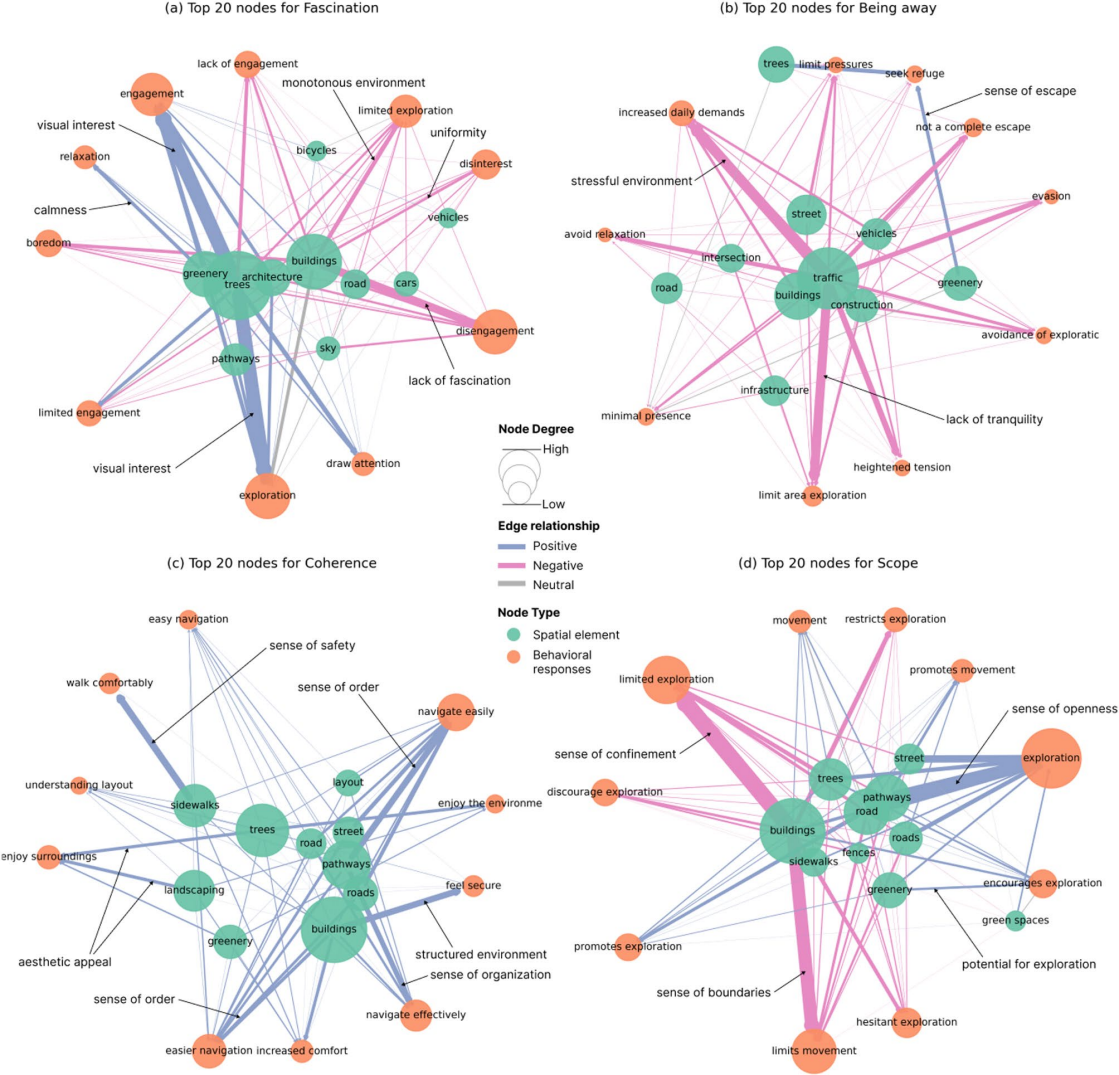
A semantic network analysis of the top 20 nodes across four restoration dimensions. (**a**) Network analysis for fascination. (**b**) Network analysis for being away. (**c**) Network analysis for coherence. (**d**) Network analysis for scope.

Next, analysis of the top 20 network nodes (10 physical elements and 10 psychological perceptions) per dimension revealed key perception-stimulus-restoration chains (Fig. 8). Within Fascination, greening elements showed positive links with multiple motivation responses, underscoring the critical role of natural elements in creating visual interest and fostering a sense of calmness. For Being Away, the pursuit of tranquility, mental breaks, and relaxation primarily originated from trees and greenery, as these elements created a sense of escape and separation. However, artificial elements (e.g., buildings) diminished the sense of escape, thereby limiting relaxation potential. Under Coherence, pavements, buildings, and greenery formed a highly integrated network, primarily eliciting positive psychological perceptions such as safety and order. The importance of spatial coherence for comprehensibility is further highlighted by the ease of navigation and comfort of walking. The Scope dimension centered on roads, buildings, and greenery, revealing spatial scale’s complex influence. While enabling positive experiences like openness, these elements could also induce negative feelings of restricted sense of exploration due to spatial constraints, reflecting the urban design’s need to balance openness with definition. These findings provide empirical evidence for urban space design and enhance understanding of perception-stimulus-restoration relationships.

**Fig. 9:**
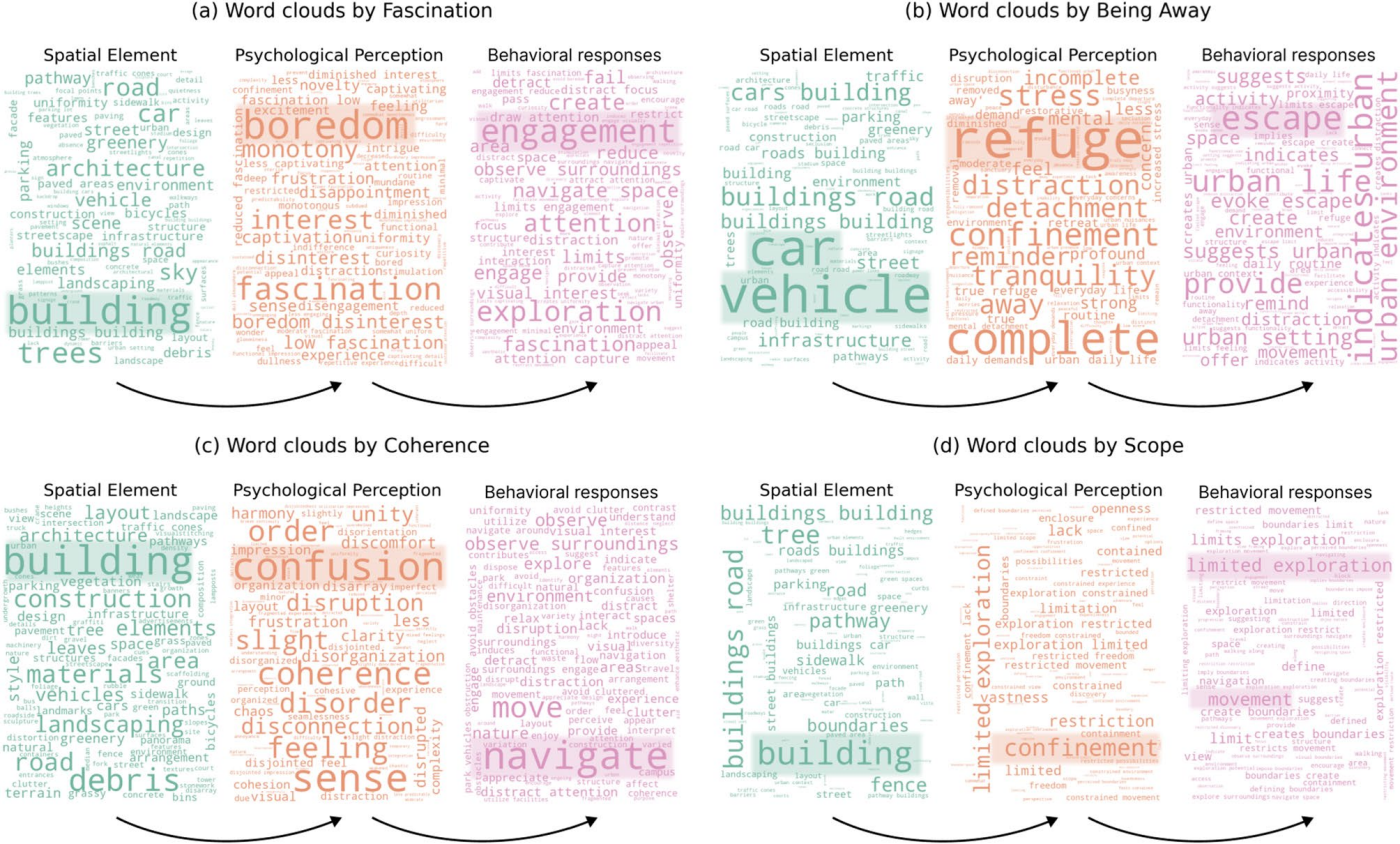
Word cloud analysis of the mechanisms behind low-quality restorative urban spaces.

To elucidate associations between environmental features and negative psychological perceptions, word clouds visualized negative relationships within each dimension, highlighting core words (Fig. 9). In Fascination, physical elements (e.g., buildings, trees, roads) formed the basic spatial framework, but boredom could be triggered, which in turn led to insufficient engagement. The Being Away dimension showed urban elements like vehicles and buildings potentially exacerbating the need for refuge. These elements, associated with urban life, often induce confinement and detachment, and it is suggested that inadequate separation opportunities reinforce the desire for complete escape. Within Coherence, the built environment was significantly linked to feelings of confusion and disruption. While navigation is a key behavioral response, environmental disorder can impede spatial cognition, implying that unclear organizational logic reduces our ability to understand and adapt to space. Finally, Scope analysis revealed physical constraints like buildings and boundaries evoking confinement, primarily manifested as restricted exploration and movement. These findings deepen our understanding of low restorative quality urban spaces and offer valuable insights for improving environmental design.

## Discussion

This study introduces a VLM-based hybrid framework for assessing the restorative quality of urban spaces. The aim is to reveal the relationship between environmental features and psychological restoration mechanisms. The core contributions of this research are as follows: First, VLMs are validated as a reliable and accurate tool for restorative assessment. Second, significant spatial heterogeneity in the distribution of restorative quality across urban spaces is revealed. Third, semantic network analysis is used to explore the intrinsic pathways connecting spatial features with psychological restoration mechanisms.

### Advantages of VLM-based methods in assessing urban space restorative quality

Our proposed VLM-based method offers clear advantages over traditional approaches when it comes to accurately and interpretably assessing the restorative quality of urban spaces. Specifically, VLMs can comprehensively and accurately capture the environmental features of urban spaces and link them to psychological restoration dimensions through multimodal inputs of images and text. For instance, existing computer vision models are limited in their detection capabilities, but VLMs can identify a broader range of meso-scale features^68^. In this process, the VLMs are assigned the role of residents. Through role prompting, they become trainable, intelligent agents that can be guided and that simulate the decision-making process of human subjects. This means that, unlike traditional methods, the restorative quality assessment process no longer maps directly from pixels to fixed scores, but instead endows VLMs with the perception-stimulus-restoration experience process from a human-centered perspective^52^. By perceiving the contextual relationships between objects within images, VLMs can directly or indirectly stimulate changes in behavior and motivation. These positive or negative psychological changes, caused by the perception-stimulus process, are categorized as potential restorative conditions. This process avoids the black-box problem inherent in traditional methods.

However, relying solely on the generalization capabilities and embedded world knowledge of VLMs is insufficient. Research by Tung, et al. ^57^ has shown that VLMs are unable to use images to predict how aesthetically pleasing a landscape will be, or to improve their predictive performance regarding landscape legibility. Our study confirms the necessity of incorporating prior knowledge. Understanding prior knowledge of evaluating urban space restorative quality enables VLMs to improve model accuracy by 106.5%. This emphasizes the importance of combining VLMs with individual evaluation data to ensure accurate results. Nevertheless, prior knowledge exhibits a threshold effect, and excessively long prior knowledge can lead to increased noise, where errors are amplified exponentially, thereby reducing the model’s ability to make judgments. This aligns with the findings of Zhang, et al. ^61^, who argue that the accuracy of perceptual prediction in VLMs plateaus as the length of prior knowledge increases once sufficient prior knowledge has been acquired. Furthermore, our study emphasizes the trend of open-source models catching up with commercial models in terms of performance. This indicates the potential for technological democratization with low barriers to entry and offers new pathways for environmental assessment in contexts where resources are limited^58^. In summary, VLMs facilitate large-scale quantitative research into subjective perception, providing an invaluable research tool for environmental psychology.

### Spatial heterogeneity of restorative quality and cognitive mechanisms

The study found that the restorative quality of urban spaces exhibits significant spatial heterogeneity. In developed areas with well-established urban green space systems, such as Nanshan and Futian, restorative quality is higher, particularly in urban parks and coastal zones. In contrast, restorative quality is typically lower in peripheral urban areas, likely due to the inferior spatial conditions resulting from rapid urbanization processes. Using semantic network analysis, we deconstructed the underlying cognitive mechanisms. High restorative areas are effective because their natural elements play a central role in the “Fascination” network, effectively alleviating directed attention fatigue through “soft fascination,” which is highly consistent with the tenets of ART^28,29^. Simultaneously, these spaces also provide the necessary “Being Away,” allowing individuals to temporarily detach from daily pressures. Interestingly, the network analysis of “Being Away” indicates that it does not stem from mere physical isolation, but can be experienced psychologically through a combination of environmental cues such as trees, sky and local greenery. This deepens our understanding of restorative experiences in everyday environments.

Crucially, the semantic network reveals the deeper meanings of “Coherence.” A tightly integrated network formed by elements like paving, buildings, and greenery is directly linked to positive psychological states such as safety, comfort, and orderliness by enhancing environmental predictability and wayfinding. This strongly corroborates that “environmental comprehensibility” is a prerequisite for restorative experience^28,34^, with research showing that easily navigable environments can reduce cognitive load and facilitate recovery from mental fatigue^76^. Finally, research has demonstrated that restorative experiences are optimized not through unlimited vastness, but by carefully designed spatial boundaries that enhance the richness of the area that can be explored^77^. This “Compatibility” between environment and user needs, as described in ART, is crucial for facilitating restoration^78^. These findings provide direct theoretical foundations for precisely guiding restorative space design.

### Practical implications for low restorative quality urban spaces

The practical significance of this study lies in providing scientific guidance for urban space optimization that transcends traditional functional considerations. First, for enhancing spatial “Fascination,” design should focus on creating interest and interactivity rather than simply adding natural elements. Diverse landscape layers and interactive installations are far more effective at stimulating exploration desire than merely increasing green areas^79^. Second, to foster “Being Away,” the design must be mindful of the stress caused by urban elements such as vehicles and dense buildings, and should strive to create micro-spaces that offer a sense of shelter^80^. This can be achieved by using plants or structures to form visual and auditory buffers, providing true refuges^81^. Third, regarding “Coherence,” design must emphasize clear wayfinding systems and logical path planning to enhance spatial legibility and avoid the sense of chaos caused by disorder^82^. Fourth, for the “Scope” dimension, the design should focus on spatial openness and explorability. By optimizing boundaries (e.g., replacing solid walls with permeable railings) and providing diverse path choices, the sense of spatial confinement can be broken, enhancing users’ positive experiences^83^. In summary, these optimization strategies based on psychological restoration mechanisms can provide residents with healthier, more comfortable living and working environments, thereby effectively promoting their physical and mental health.

### Limitations

However, this study still has some limitations. First, the sample size of human annotations is limited, which may affect the transferability of the research findings. Furthermore, we did not investigate variations across different demographic strata (such as gender differences in urban space restorative quality assessment). Second, street view images have certain shortcomings, such as limited perspectives, difficulty capturing seasonal changes, and challenges in reflecting dynamic environments^84^. These factors may influence VLMs assessment results. Indeed, some studies have confirmed the impact of multi factors like nature sounds and smell on psychological restoration^85,86^. Therefore, subsequent research needs to incorporate multiple data sources, such as satellite imagery and sound, to improve the accuracy of assessment results. Finally, VLMs may have potential biases, especially when handling different cultural backgrounds and individual differences, which could lead to misinterpretations of local cultural landscapes^87^. Future research needs to further optimize the prior knowledge and algorithms of VLMs to reduce bias impacts and enhance model fairness and reliability.

## Conclusion

This study proposes a novel VLM-based hybrid framework for measuring restorative quality of urban spaces. Experimental results demonstrate that our approach significantly improves prediction accuracy (R²) by 0.535 compared to traditional ML methods. This substantial enhancement primarily stems from the infusion of prior knowledge integrating both subjective and objective information. Through large-scale empirical investigation, we reveal the characteristic spatial heterogeneity of urban space restorative quality, identifying significant variations in the impact of different areas and spatial elements on restorative outcomes. Furthermore, by leveraging semantic network analysis, we are able to systematically reveal the perception-stimulus-restoration mechanism that underlies restorative urban environments. This provides new insights into the psychological effects of environments on humans. Finally, for areas of low restorative quality, we propose actionable optimization strategies based on the four restorative dimensions. These strategies offer a scientific basis for enhancing urban environmental quality. The presented methodology and findings offer significant theoretical and practical value for the development of sustainable cities and environmental psychology research.

## Supplementary Information

Below is the link to the electronic supplementary material.

Supplementary Material 1

## Data Availability

The datasets and code generated in this study are available at https://github.com/MMHHRR/VLM\_restorative-quality.

## Footnotes

1
http://psy.china.com.cn.
