# Supplementary material for: Measuring the psychological restorative quality of urban spaces: a vision language model-based method
Source: Sci Rep. 2026 Apr 5;16:16534. doi: 10.1038/s41598-026-43360-8 (PMC13216355; doi:10.1038/s41598-026-43360-8)
Supplement: Supplementary file 1 — Supplementary Material 1 [file 41598_2026_43360_MOESM1_ESM.pdf]

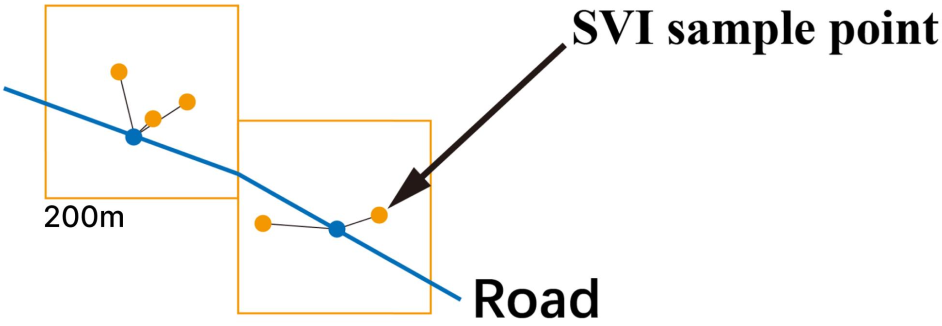

Figure S1. Sample collection. We firstly used a quantitative method to collect SVIs samples based on grid units (200 × 200 meter of each grid) generating from road network (Dhakal et al.,2000). This method ensures that the samples are as evenly distributed as possible in space, resulting in a total of 566 sample points that include the corresponding SVIs (n = 566).

[1] Dhakal, A.S., Amada, T., Aniya, M., et al., 2000. Landslide hazard mapping and its evaluation using gis: an investigation of sampling schemes for a grid-cell based quantitative method. Photogrammetric engineering and remote sensing 66, 981–989.

Table S1. Restorative quality indicators (PRS-11).

Restorative quality	Function	Questions
Being away	Elimination of everyday distractors.	BQ1: Places like that are a refuge from nuisances<br><br>BQ2: To get away from things that usually demand my attention I like to go to places like this<br><br>BQ3: To stop thinking about the things that I must get done I like to go to places like this
Coherence	Sufficient connectedness makes it possible to build a mental map and make sense of the environment.	CQ1: There is a clear order in the physical arrangement of places like this<br><br>CQ2: In places like this it is easy to see how things are organized<br><br>CQ3: In places like this everything seems to have its proper place
Scope	Sufficient scope building a mental map worthwhile by facilitating curiosity and a desire to be involved in the environment.	SQ1: That place is large enough to allow exploration in many directions<br><br>SQ2: In places like that there are few boundaries to limit my possibility for moving about
Fascination	Reducing mental fatigue by shifting from voluntary to involuntary attention.	FQ1: Places like that are fascinating<br><br>FQ2: In places like this my attention is drawn to many interesting things<br><br>FQ3: In places like this it is hard to be bored

Table S2. Prompt engineering.

Message template

'''

You are a university student participating in a campus environment assessment. Carefully analyze the provided image to evaluate psychological restoration quality. Provide step-by-step reasoning before final scoring.

Evaluate the space using Perceived Restorativeness Scale (PRS)-11 (0-1 scale) across four dimensions. **1 = Strongly agree, 0 = Strongly disagree**. Base all judgments strictly on image features and prior knowledge

Prior Knowledge:\n{RAG}.

Evaluation Dimensions:

1. Fascination
- Places like that are fascinating
- In places like this my attention is drawn to many interesting things
- In places like this it is hard to be bored

2. Being Away
- Places like that are a refuge fom nuisances
- To get away from things that usually demand my attention I like to go to places like this
- To stop thinking about the things that I must get done I like to go to places like this

3. Coherence
- There is a clear order in the physical arrangement of places like this
- In places like this it is easy to see how things are organised
- In places like this everything seems to have its proper place

4. Scope
- That place is large enough to allow exploration in many directions
- In places like that there are few boundaries to limit my possibility for moving about

Please provide follow information ONLY:

1. Average score of all dimension (Scale 0-1 with 5 decimal places)
2. Explanation for each PRS dimension (limited to 200 words)

You MUST output JSON format ONLY:

```
{{
  "score": 0.75,
  "reason": {{
     "fascination": XXX,
     "being_away": XXX,
     "coherence": XXX,
     "scope": XXX,
  }}
}}
```

'''

Table S3. Statistical summary of dataset based on PRS-11.

Dimension	Count	Mean	Std.	95% CI
**Pixel-level**				
hue	566	0.211	0.052	(0.204, 0.218)
saturation	566	0.200	0.073	(0.190, 0.210)
edges	566	0.110	0.037	(0.105, 0.115)
threshold	566	0.329	0.137	(0.310, 0.348)
brightness	566	0.587	0.092	(0.574, 0.600)
**Perceptual-level**	566			
beauty	566	0.328	0.080	(0.317, 0.339)
boredom	566	0.403	0.061	(0.395, 0.412)
depressing	566	0.481	0.055	(0.473, 0.489)
liveliness	566	0.347	0.071	(0.338, 0.357)
safety	566	0.363	0.057	(0.355, 0.371)
wealth	566	0.437	0.076	(0.427, 0.448)
**Semantic-level**	566			
road	566	0.235	0.120	(0.201, 0.301)
sidewalk	566	0.018	0.028	(0.014, 0.022)
building	566	0.092	0.087	(0.080, 0.104)
wall	566	0.005	0.012	(0.004, 0.007)
fence	566	0.016	0.033	(0.011, 0.020)
traffic light	566	0.004	0.005	(0.003, 0.005)
traffic sign	566	0.002	0.005	(0.001, 0.002)
vegetation	566	0.242	0.122	(0.225, 0.259)
terrain	566	0.081	0.073	(0.071, 0.091)
sky	566	0.235	0.100	(0.221, 0.248)
person	566	0.004	0.011	(0.003, 0.006)
rider	566	0.003	0.005	(0.002, 0.003)
car	566	0.016	0.019	(0.013, 0.019)
